# Efficacy and Safety of Low-Dose Nab-Paclitaxel Plus Tislelizumab in Elderly Patients With Previously Treated Metastatic Non-Small Cell Lung Cancer

**DOI:** 10.3389/fonc.2022.802467

**Published:** 2022-03-17

**Authors:** Wenyu Zhu, Qian Geng, Haoliang Peng, Zhihui Jin, Dongqing Li, Xiaolin Pu, Ge Wang, Hua Jiang

**Affiliations:** ^1^ Cancer Center, The Affiliated Changzhou No. 2 People’s Hospital of Nanjing Medical University, Changzhou, China; ^2^ Department of Oncology, The First Affiliated Hospital of Nanjing Medical University, Nanjing, China; ^3^ Department of Oncology, Graduate School of Dalian Medical University, Dalian, China

**Keywords:** low-dose, chemotherapy, immunotherapy, elderly, non-small cell lung cancer

## Abstract

The combination of immunotherapy and chemotherapy has a synergic effect in non-small cell lung cancer (NSCLC). However, the elderly are often excluded from clinical trails due to their poor health status and more comorbidities. We sought to assess the efficacy and safety of low-dose nanoparticle albumin-bound paclitaxel (nab-paclitaxel) plus tislelizumab (an anti-PD-1 antibody) in elderly patients with advanced NSCLC. In this phase 2 clinical trail, eligible patients were those aged ≥65 years with metastatic NSCLC who had disease progression after treatment with ≥1 line of chemotherapy or targeted therapy. Patients with epidermal growth factor receptor (*EGFR)* or anaplastic lymphoma kinase (*ALK)* variations were eligible if they demonstrated disease progression after treatment with ≥1 corresponding inhibitor. Primary endpoints were progression-free survival and safety/tolerability. Secondary endpoints included objective response rate and overall survival. Among 29 patients enrolled from May 2019 through August 2020, 21 (72.4%) had adenocarcinoma, 17 (58.6%) had a performance status of 2, 8 (27.6%) had asymptomatic brain metastases, and 13 (44.8%) had *EGFR*/*ALK* variations. As of the data cutoff point on April 1, 2021, median progression-free survival and overall survival were 9.5 months and 16.5 months, respectively. Ten patients achieved a partial response (objective response rate of 34.5%). Seventeen (58.6%) patients had ≥1 treatment-related adverse event, with grade 3 events seen in 3 patients (10.3%). The most common adverse events were fatigue (20.7%), fever (17.2%), abnormal liver function (17.2%), and rash (17.2%). These results suggest that low-dose nab-paclitaxel plus tislelizumab is well tolerated and effective in elderly patients with advanced NSCLC, including those with *EGFR*/*ALK* variations.

## Introduction

Lung cancer, which accounts for 11.6% of all new cancer cases worldwide, is the leading cause of cancer-related mortality, with a 5-year survival rate of 4% to 17% (1). Approximately 80% to 85% of lung cancer cases are classified as non-small cell lung cancer (NSCLC) (2, 3). Conventional chemotherapy used to be the standard treatment option for patients with advanced NSCLC, but the 5-year survival rate with this treatment was only 5% (4). Targeted therapy has a good therapeutic outcome in patients with specific molecular subtypes such as epidermal growth factor receptor (*EGFR)* mutation or anaplastic lymphoma kinase (*ALK)* positive, but eventual drug resistance is unavoidable in most cases, and many patients do not have the gene mutations targeted by these specific therapies. With the development of immunotherapy, immune checkpoint inhibitors such as programmed cell death-1 (PD-1) and programmed cell death ligand-1 (PD-L1) antibodies have gradually become the standard treatment in patients with advanced NSCLC (5). Several studies, such as KEYNOTE-189, have also suggested that the combination of immunotherapy with standard-dose chemotherapy can have a synergic effect in NSCLC, leading to improved survival outcomes (6–8).

Elderly patients represent a high proportion of the NSCLC population (9). Because these patients are more frequently frail and have more comorbidities, the risks associated with full dose of chemotherapy are higher, thus limiting the treatment options available (10). As a result, they are often excluded from clinical trials. Additionally, no consensus has been reached regarding treatment recommendations or risk/benefit ratios. For these patients, suitable treatments that can effectively control the disease while minimizing the risk of adverse reactions are urgently needed.

Nanoparticle albumin-bound paclitaxel (nab-paclitaxel) is a novel nanoparticulate formulation of paclitaxel bound to human serum albumin that has been found to improve the therapeutic index of paclitaxel (11). In a phase 3 trial, first-line treatment with nab-paclitaxel in combination with carboplatin was effective and well tolerated in patients aged ≥70 years with advanced NSCLC (12). Earlier phase 2 trials also reported good efficacy and safety with nab-paclitaxel monotherapy as second-line treatment for patients with NSCLC who had disease progression after platinum-based chemotherapy (13–15). Nab-paclitaxel may therefore be a useful alternative for elderly patients with advanced NSCLC.

As mentioned earlier, a combination of immunotherapy and chemotherapy may improve outcomes in patients with NSCLC. Tislelizumab, a humanized anti-PD-1 monoclonal antibody, is engineered to minimize binding to FcγR on macrophages to abrogate antibody-dependent cellular phagocytosis, a mechanism of T-cell clearance and potential resistance to anti-PD-1 therapy (16, 17). In studies, tislelizumab has been well tolerated with no unexpected safety concerns in patients with advanced NSCLC (18). Regardless of ethnicity and PD-L1 expression, promising efficacy has been observed in heavily pretreated patients with NSCLC who received tislelizumab as monotherapy and in untreated patients with NSCLC who received tislelizumab in combination with chemotherapy (18).

Because elderly patients with NSCLC have more complications, weaker bone marrow function, and lower tolerance to treatment, we sought to determine whether it would be possible to reduce the dose of chemotherapy and still achieve good outcomes in these patients by combining this treatment with tislelizumab. In this study, we therefore evaluated the efficacy and safety of combination therapy with low-dose nab-paclitaxel and tislelizumab in elderly patients with metastatic NSCLC who had previously been treated with platinum-based chemotherapy or targeted therapy.

## Methods

### Study Design

This single-arm, open-label, investigator-initiated phase 2 prospective study was performed at the Affiliated Changzhou No. 2 People’s Hospital of Nanjing Medical University in Changzhou, China. The study was designed to evaluate the efficacy and safety of treatment with tislelizumab plus low-dose nab-paclitaxel in patients aged ≥65 years who had metastatic NSCLC with disease progression after platinum-based chemotherapy or targeted therapy. The study was approved by the hospital’s ethics committee and was conducted in accordance with the principles of the Declaration of Helsinki. All patients included in the study provided written informed consent.

### Patients

From May 2019 through August 2020, we recruited patients aged ≥65 years with histologically confirmed metastatic NSCLC and with ≥1 measurable lesion who had demonstrated disease progression after treatment with platinum-based chemotherapy or targeted therapy. Patients with *EGFR* or *ALK* variations were eligible to participate if they had demonstrated disease progression after treatment with ≥1 approved corresponding inhibitor. Patients with asymptomatic brain metastases were also eligible, regardless of Eastern Cooperative Oncology Group performance status (PS) and PD-L1 expression. Patients who received previous treatment with drugs specifically targeting checkpoint pathways were excluded from the study.

### Procedures

Participants included in the study received nab-paclitaxel (130 mg/m^2^), day 1, every 3 weeks) for ≤6 cycles plus tislelizumab (200 mg, day 2, every 3 weeks), followed by maintenance therapy with tislelizumab (200 mg, every 3 weeks) until confirmed disease progression, death, or unacceptable toxicity.

### Study Assessments

The primary endpoints were progression-free survival (PFS) and safety/tolerability. Secondary endpoints included objective response rate (ORR), disease control rate (DCR), and overall survival (OS). Tumor responses were assessed according to the Response Evaluation Criteria in Solid Tumors (RECIST 1.1) and included complete response (CR), partial response (PR), stable disease (SD), and progressive disease (PD). ORR was defined as the sum of CR and PR. DCR was defined as the sum of CR, PR, and SD. PFS was defined as the time from the first day of nab-paclitaxel until disease progression or death by any cause whichever occurred first. OS was defined as the time from the date of trial enrollment to death or last follow-up. Duration of response (DoR) was defined as the time from first RECIST response to disease progression in patients who demonstrated at least a PR. We assessed response to treatment every 6 weeks using CT based on RECIST 1.1 criteria. Treatment-related adverse events (TRAEs) were monitored and graded using the National Cancer Institute Common Terminology Criteria for Adverse Events (version 4.0). The data cutoff was April 1, 2021.

### Statistical Analysis

Continuous variables were summarized as arithmetic means or medians with standard deviations (SDs) or percentiles for descriptive purposes, and categorical variables were presented as frequencies and percentages. χ^2^ tests or Fisher exact tests were used to assess the statistical significance of categorical variables. ORR was presented as a percentage with 95% confidence intervals (CIs). PFS, DoR, and OS were analyzed using the Kaplan-Meier method. Subgroups were compared using the log-rank test. For survival curve analysis, all variables were dichotomized according to their medians. Statistical significance was defined as *P*<0.05 using a 2-tailed test. All statistical analyses were performed using SPSS version 19.0.

## Results

### Patients

From May 2019 through August 2020, a total of 30 cases of patients with NSCLC were initially identified as eligible for study inclusion. Among these patients, 1 declined to participate; therefore, a total of 29 patients (mean age, 70.3 ± 4.1 years) were enrolled in the study. Among the 29 recruited patients, 14 (48.3%) were men, 12 (41.4%) were current/former smokers, 17 (58.6%) had a PS of 2, and 8 (27.6%) had asymptomatic brain metastases. In total, 13 patients (44.8%) with *EGFR*/*ALK* variations were included in the study (11 with *EGFR* variations and 2 with *ALK* variations). The most common histologic diagnosis was adenocarcinoma, accounting for 21 (72.4%) patients. The expression of PD-L1 was available for 18 (62.1%) patients; of these patients, 8 demonstrated PD-L1 expression <1%; 7, 1% to 10%, and 3, >10% (**Table 1**).

**Table 1:** Demographic and baseline characteristics of study patients (N = 29).

Mean age ± SD, years	70.3 ± 4.1
Eastern Cooperative Oncology Group PS, n (%)	
0	1 (3.4)
1	11 (37.9)
2	17 (58.6)
Sex, n (%)	
Male	14 (48.3)
Female	15 (51.7)
Pathological type, n (%)	
Squamous cell carcinoma	8 (27.6)
Adenocarcinoma	21 (72.4)
Smoking status, n (%)	
Current or former smoker	12 (41.4)
Never smoker	17 (58.6)
Metastatic sites, n (%)	
Brain	8 (27.6)
Liver	7 (24.1)
Bone	6 (20.7)
Lung	14 (48.3)
Previous lines of therapy, n (%)	
1	13 (44.8)
2	12 (41.4)
≥3	4 (13.8)
PD-L1 % expression in tumor cells, n (%)	
<1%	8 (27.6)
1%-10%	7 (24.1)
>10%	3 (10.3)
Not available	11 (37.9)
Oncotarget variation, n (%)	
* EGFR* variations	11 (37.9)
* ALK* variations	2 (6.9)

SD, standard deviation; PS, performance status; PD-L1, programmed cell death ligand-1.

Patients had received 1 (13/29, 44.8%), 2 (12/29, 41.4%), or ≥3 (4/29, 13.8%) lines of therapy previously. All 16 patients without driver gene mutations had received platinum-based chemotherapy, most commonly pemetrexed plus carboplatin. All 11 patients with *EGFR* variations had received a first- or second-generation *EGFR* tyrosine kinase inhibitor (afatinib, gefitinib, erlotinib, or icotinib). Both of the patients with *ALK* variations had received crizotinib.

The mean number of nab-paclitaxel chemotherapy cycles was 4.6 ± 1.7 among study patients. Fifteen of the 29 patients (51.7%) received treatment for 6 cycles. As of April 1, 2021, the median follow-up was 14.0 months (95% CI, 8.5-19.6); on this date, 1 patient was still receiving treatment with tislelizumab. The most common reason for treatment discontinuation was progressive disease (n = 22; 75.9%).

### Efficacy

**Figure 1:**
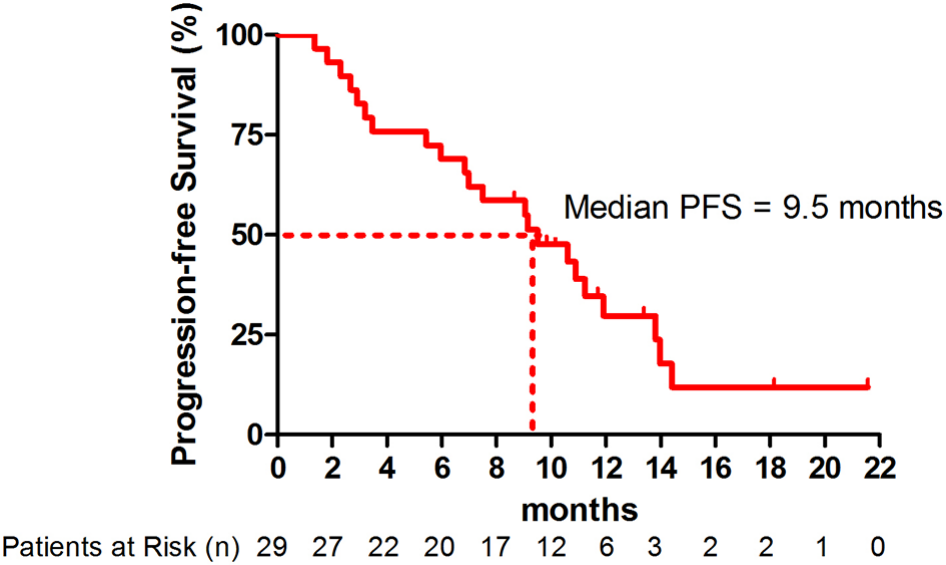
Kaplan-Meier curve for progression-free survival (PFS) (n = 29).

At the point of data cutoff, 22 patients (75.9%) had disease progression. The overall median PFS was 9.5 months (95% CI, 5.8-13.2 months) (**Figure 1**). The median PFS was 10.9 months (95% CI, 8.6-13.2 months) in patients with adenocarcinoma versus 3.2 months (95% CI, 0-10.4 months) in patients with squamous cell carcinoma (hazard ratio [HR], 0.63; 95% CI, 0.24-1.62; *P*=0.33) (**Figure S1** and **Table S1**). The median PFS was significantly higher in patients with nonsquamous NSCLC with *EGFR/ALK* wild type (11.9 months; 95% CI, 9.3-14.5 months) than in those with *EGFR/ALK* variations (7.0 months; 95% CI. 3.5-10.5 months; HR, 0.56; 95% CI, 0.33-0.97; *P*=0.032) (**Figure S2**).

**Figure 2:**
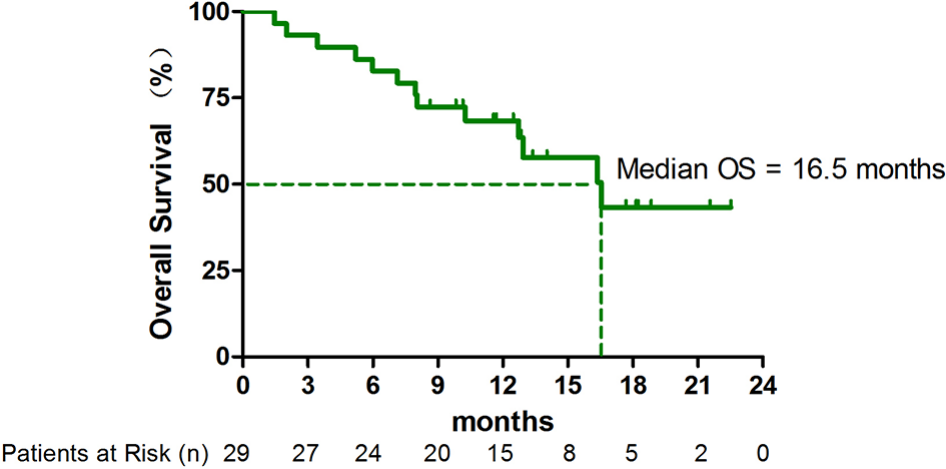
Kaplan-Meier curve for overall survival (OS) (n = 29).

As of April 1, 2021, 13 (44.8%) patients had died. The median OS was 16.5 months (95% CI, 10.8-22.3 months) (**Figure 2**). In subgroup analysis, the median OS in patients with adenocarcinoma with *EGFR/ALK* wild type has not yet been reached (**Table S1**).

**Figure 3:**
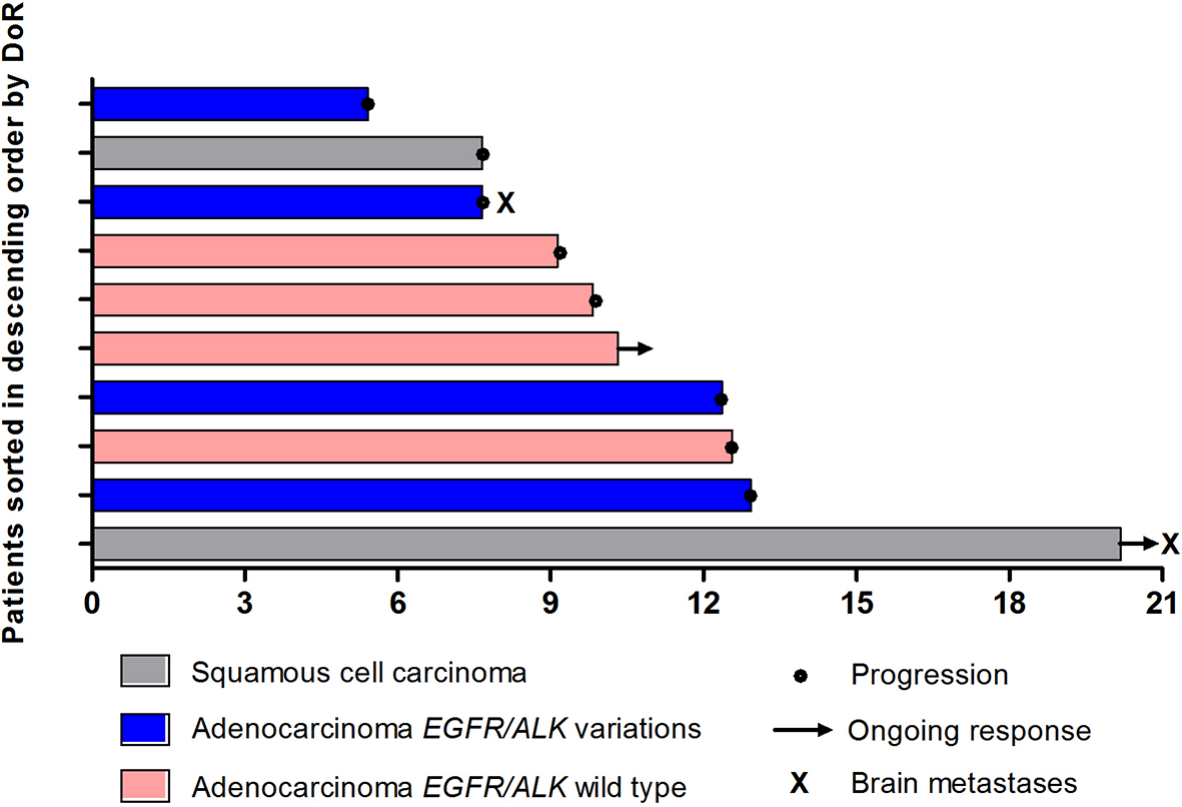
Duration of response (DoR) (n = 10).

In total, 10 patients achieved PR, leading to an ORR of 34.5% (95% CI, 17.9%-54.3%) (**Table 2**). As the point of data cutoff, the median duration of these responses was 9.8 months (95% CI, 5.4–14.3 months), with 2 response ongoing (**Figure 3**). The ORR was 38.1% (95% CI, 18.1%-61.6%) in patients with adenocarcinoma versus 25.0% (95% CI, 3.2%-65.1%; *P* = 0.419) in patients with squamous cell carcinoma (**Table 2**). In patients with nonsquamous NSCLC, the ORR was 50.0% (95% CI, 15.7%-84.3%) in patients with *EGFR/ALK* wild type versus 30.8% (95% CI, 9.1%-61.4%) in those with *EGFR/ALK* variations (*P* =0 .336) (**Table 2**).

**Table 2:** Response to treatment.

Response	All patients (N = 29)	Patients with squamous cell carcinoma (n = 8)	Patients with adenocarcinoma,* EGFR/ALK* variations (n = 13)	Patients with adenocarcinoma, *EGFR/ALK* wild type (n = 8)
Best overall response, n (%)				
Complete response	0	0	0	0
Partial response	10 (34.5)	2 (25.0)	4 (30.8)	4 (50.0)
Stable disease	15 (51.7)	3 (37.5)	8 (61.5)	4 (50.0)
Progressive disease	4 (13.8)	3 (37.5)	1 (7.7)	0
Objective response rate, n (%)	10 (34.5)	2 (25.0)	4 (30.8)	4 (50.0)
Disease control rate, n (%)	25 (86.2)	5 (62.5)	12 (92.3)	8 (100.0)

**Figure 4:**
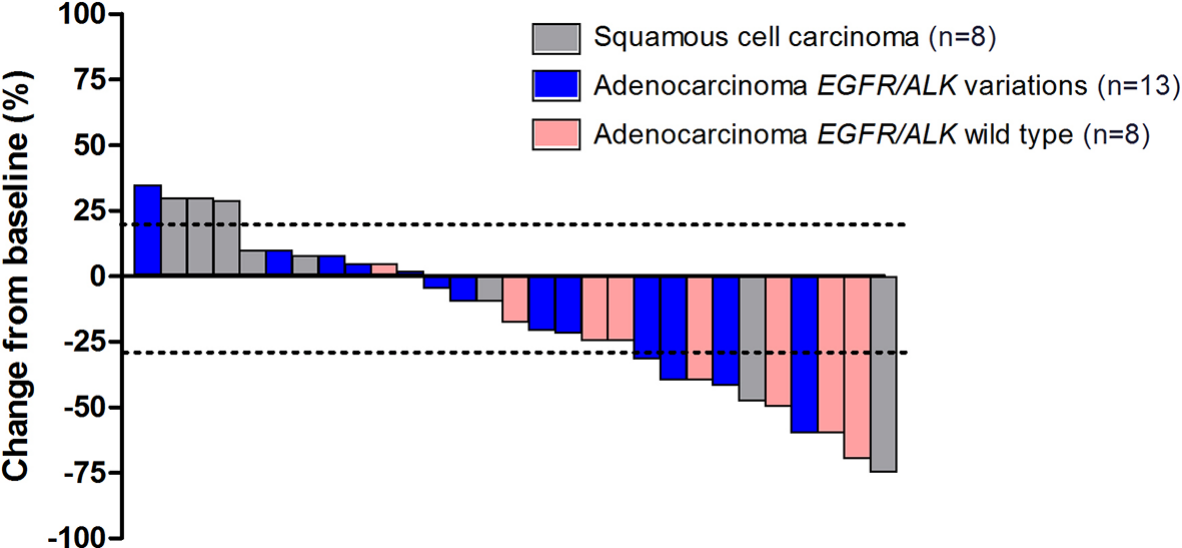
The maximum percentage reduction from baseline in sum of target lesions based on histology and *EGFR/ALK* status (n = 29).

Overall, disease control was reported in 25 patients (86.2%) (**Table 2**). Additionally, among 8 patients with asymptomatic brain metastases, PR was achieved in 2 patients, and the DCR was 100.0%. Eighteen patients overall (62.1%) had a decrease in tumor size from baseline (median change, −18%) (**Figure 4**).

### Safety

A total of 17 patients (58.6%) suffered at least 1 TRAE, with 3 patients (10.3%) experiencing a grade 3 adverse event (**Table 3**). No grade 4 adverse events were reported. The most common TRAEs were fatigue (6/29; 20.7%), fever (5/29; 17.2%), abnormal liver function (5/29; 17.2%), rash (5/29; 17.2%), and numbness (4/29; 13.8%). Only 1 (3.4%) patient discontinued treatment because of an adverse event (immune-related pneumonitis).

**Table 3:** Incidence of treatment-related adverse events.

Treatment-related adverse events	Any grade, n (%)	Grade 1-2, n (%)	Grade ≥3, n (%)
Fatigue	6 (20.7)	5 (17.2)	1 (3.4)
Fever	5 (17.2)	3 (10.3)	2 (6.9)
Abnormal liver function	5 (17.2)	4 (13.8)	1 (3.4)
Rash	5 (17.2)	5 (17.2)	0
Numbness	4 (13.8)	4 (13.8)	0
Loss of appetite	2 (6.9)	2 (6.9)	0
Leukopenia	2 (6.9)	1 (3.4)	1 (3.4)
Anemia	1 (3.4)	1 (3.4)	0
Hyponatremia	1 (3.4)	1 (3.4)	0
Hypothyroidism	1 (3.4)	1 (3.4)	0
Skin pigmentation	1 (3.4)	1 (3.4)	0
Diarrhea	1 (3.4)	1 (3.4)	0
Immune-related pneumonitis	1 (3.4)	1 (3.4)	0

### Follow Up

After the disease progressed, 16/22 (72.7%) patients received the follow-up treatment, among which 2 (12.5%) received next-generation *EGFR*/*ALK* targeted drugs, 7 (43.75%) received mono antiangiogenic agents, 4 (25.0%) received chemotherapy plus antiangiogenic agents, the last 3 (18.75%) received immunotherapy plus antiangiogenic agents. In general, antiangiogenic targeted therapy played a major role in the posterior treatment.

## Discussion

Immunotherapy can dramatically improve clinical outcomes in patients with advanced NSCLC without targetable genetic alterations. Additionally, research has shown that apart from chemotherapy’s direct cytotoxic activity, this treatment exerts immune-potentiating effects such as increasing the immunogenicity of tumor cells, inhibiting negative immune signals, and altering the immune microenvironment (19–21). Chemotherapy has also been shown to induce PD-L1 expression on tumor cells (22, 23). Thus, the combination of immunotherapy and chemotherapy has a synergistic effect. Because elderly patients are less able to tolerate the toxicities associated with standard-dose chemotherapy, we sought to assess the combination of low-dose chemotherapy and immunotherapy in elderly patients with previously treated metastatic NSCLC. We found that low-dose nab-paclitaxel plus immunotherapy [with chemotherapy administered before immunotherapy to enhance antigen liberation and increase the synergistic effects (24, 25)] was safe and effective in these patients.

To our knowledge, this is the first clinical trial to prospectively report that the combination of low-dose nab-paclitaxel and tislelizumab is well tolerated and leads to promising outcomes in elderly patients with advanced NSCLC, including patients with *EGFR* or *ALK* variations. A previous study demonstrated a PFS of 7.6 months and an ORR of 74.8% in patients who received tislelizumab plus nab-paclitaxel and carboplatin as first-line treatment for advanced squamous NSCLC, whereas patients treated with paclitaxel plus carboplatin alone had a shorter PFS, highlighting the possible synergistic effect between chemotherapy and immunotherapy (26). Nab-paclitaxel monotherapy has previously been found to be efficacious as second-line treatment for patients with NSCLC, as indicated by ORRs of 14.5% to 31.7%, DCRs of 65.5% to 71.9%, median PFS values of 3.9 to 5.1 months, and median OS values of 9.9 to 13.0 months (13–15). Similar results have been seen with other immunotherapy and chemotherapy combinations. For instance, in a randomized phase 2 trial, the addition of pembrolizumab to docetaxel was found to significantly improve PFS when compared with docetaxel alone (9.5 months vs 3.9 months; HR, 0.24) in patients with disease progression after platinum-based chemotherapy (24). The ORR was also improved with combination therapy (42.5% vs 15.8%).

Though the ORR in our study was not as high as in this previous study, the results are still noteworthy in consideration of the poor physical condition of patients as around 50% of them had a PS of 2. Meanwhile, it translated into a PFS benefit nonetheless, representing a longer duration of response, suggesting that low-dose chemotherapy combined with immunotherapy can also produce good synergy effects with tolerable side effects especially for those patients with poor physical conditions. The molecular mechanism of this potential synergistic effect has not yet been fully explained; however, our C57BL/6 mouse lung cancer model study demonstrated an increase in the expression of PD-L1 and CD8^+^ T lymphocytes in the immune microenvironment with combination therapy of anti-PD-1 antibody plus chemotherapy, especially in the group of half-dose chemotherapy and anti-PD-1 antibody (data not shown), which may be a factor. One possible explanation is that low-dose chemotherapy was inclined to induce the immunogenicity of tumor cells rather than killing tumor cells. Therefore, low-dose chemotherapy not only maintains antitumor efficacy but also reduces the adverse effects of combination therapy.

One interesting finding in this study was the efficacy of low-dose nab-paclitaxel plus tislelizumab in patients with *EGFR/ALK* variations, who have generally been underrepresented in previous clinical trials (27, 28). It is well known that the frequency of *EGFR* variations in the Chinese population is relatively high (29), so immunotherapy options must be considered for patients with these variations whose disease is resistant to *EGFR* tyrosine kinase inhibitors. A total of 13 patients (44.8%) with *EGFR/ALK* variations were included in this study. In this patient subgroup, the PFS and ORR were lower than those of patients with *EGFR/ALK* wild type, but patients with *EGFR/ALK* variations still demonstrated improvements in PFS and ORR with this treatment combination. Previous studies included only a small number of patients with *EGFR* variations (24, 30); however, these studies did demonstrate that the addition of immunotherapy (atezolizumab or pembrolizumab) significantly improves PFS in patients with genetically altered *EGFR* in advanced nonsquamous NSCLC. Taken together, these results suggest that large trials should be conducted to further assess the use of combination therapy after treatment with tyrosine kinase inhibitors in patients with nonsquamous NSCLC with *EGFR/ALK* variations.

Another interesting finding in this study was the efficacy of this treatment regimen in patients with brain metastases, a population with limited available data. Metastases to the brain, which manifest as neurological disorders, occur in 18% to 61% of patients with lung cancer and are associated with a poor prognosis (31–33). The role of chemotherapeutic agents in these patients is limited by the blood–brain barrier, but the introduction of targeted therapies and immune checkpoint inhibitors has radically changed the treatment algorithm for this patient population (34). Despite these changes, most previous studies have recruited only a small percentage of patients with brain metastases, which is not representative of the overall population. In our study, 8 patients with asymptomatic brain metastases were included, 2 of whom achieved PR. The DCR in this subgroup was 100%, highlighting the efficacy of this treatment combination. These data support the need for well-designed larger trials exploring combination therapy in this patient subgroup.

Because elderly patients have decreased organ function and a higher incidence of comorbidities, they are more likely to experience toxicities related to chemotherapy, making the treatment of these patients challenging (35). In this study, TRAEs were reported in 17 of 29 patients (58.6%), a lower rate than has been reported in previous studies of combination therapy, even with the inclusion of patients with a PS of 2. In addition, most of the TRAEs were grade 1 or 2 and were manageable; no grade 4 adverse events were reported.

This study had several limitations, key among them the small number of patients who met the eligibility criteria within the study period. This led to insufficient statistical power for the data analysis. We will continue to enroll patients to expand the sample size. Second, this was a single-arm study; comparisons with full-dose chemotherapy plus immunotherapy or with immunotherapy alone were not made. Third, data were missing regarding PD-L1 expression and tumor mutation burden for some of the study patients, so we were unable to evaluate these factors as biomarkers. Despite these limitations, this is the first trial to demonstrate that low-dose nab-paclitaxel plus tislelizumab is safe and effective in elderly patients with metastatic NSCLC when used as second- or later-line treatment, even though half of the patients had a PS of 2 and many had NSCLC with *EGFR/ALK* variations. Indeed, this trial population may be representative of the real-world patient population, as it included patients with higher PS scores, patients with asymptomatic brain metastases, patients with *EGFR/ALK* variations, and patients with various levels of PD-L1 expression. Further randomized controlled trials, preferably in large patient populations, are needed to confirm these results. Also we can envisage that maybe this combination can even be a valuable first-line option in patients not candidate to platinum-based chemotherapy. Randomized clinical trials evaluating this combination could be run in the setting of first-line for elderly NSCLC patients.

In summary, we found that for elderly patients with advanced NSCLC, including those with high PS scores, brain metastases, or *EGFR/ALK* variations, combination therapy with low-dose nab-paclitaxel and tislelizumab is safe and has synergistic effects. Our results raise the possibility of using this treatment regimen clinically in this patient population with the goal of prolonging patient survival.

## Data Availability Statement

The original contributions presented in the study are included in the article/**Supplementary Material**. Further inquiries can be directed to the corresponding author.

## Ethics Statement

The studies involving human participants were reviewed and approved by Changzhou No.2 People’s Hospital. The patients/participants provided their written informed consent to participate in this study. Written informed consent was obtained from the individual(s) for the publication of any potentially identifiable images or data included in this article.

## Author Contributions

All authors made a significant contribution to the work reported, whether that is in the conception, study design, execution, acquisition of data, analysis and interpretation, or in all these areas, took part in drafting, revising or critically reviewing the article, gave final approval of the version to be published, have agreed on the journal to which the article has been submitted, and agree to be accountable for all aspects of the work.

## Funding

This study was supported by the Research Fund from Chinese Society of Clinical Oncology (Grant No. Y-sy2018-030), and the Young Scientists Foundation of Changzhou No.2 People’s Hospital (2018K004).

## Conflict of Interest

The authors declare that the research was conducted in the absence of any commercial or financial relationships that could be construed as a potential conflict of interest.

## Publisher’s Note

All claims expressed in this article are solely those of the authors and do not necessarily represent those of their affiliated organizations, or those of the publisher, the editors and the reviewers. Any product that may be evaluated in this article, or claim that may be made by its manufacturer, is not guaranteed or endorsed by the publisher.
