# Supplementary material for: Efficacy and Safety of Low-Dose Nab-Paclitaxel Plus Tislelizumab in Elderly Patients With Previously Treated Metastatic Non-Small Cell Lung Cancer
Source: Front Oncol. 2022 Mar 17;12:802467. doi: 10.3389/fonc.2022.802467 (PMC8968868; doi:10.3389/fonc.2022.802467)
Supplement: Supplementary file 1 [file DataSheet_1.docx]

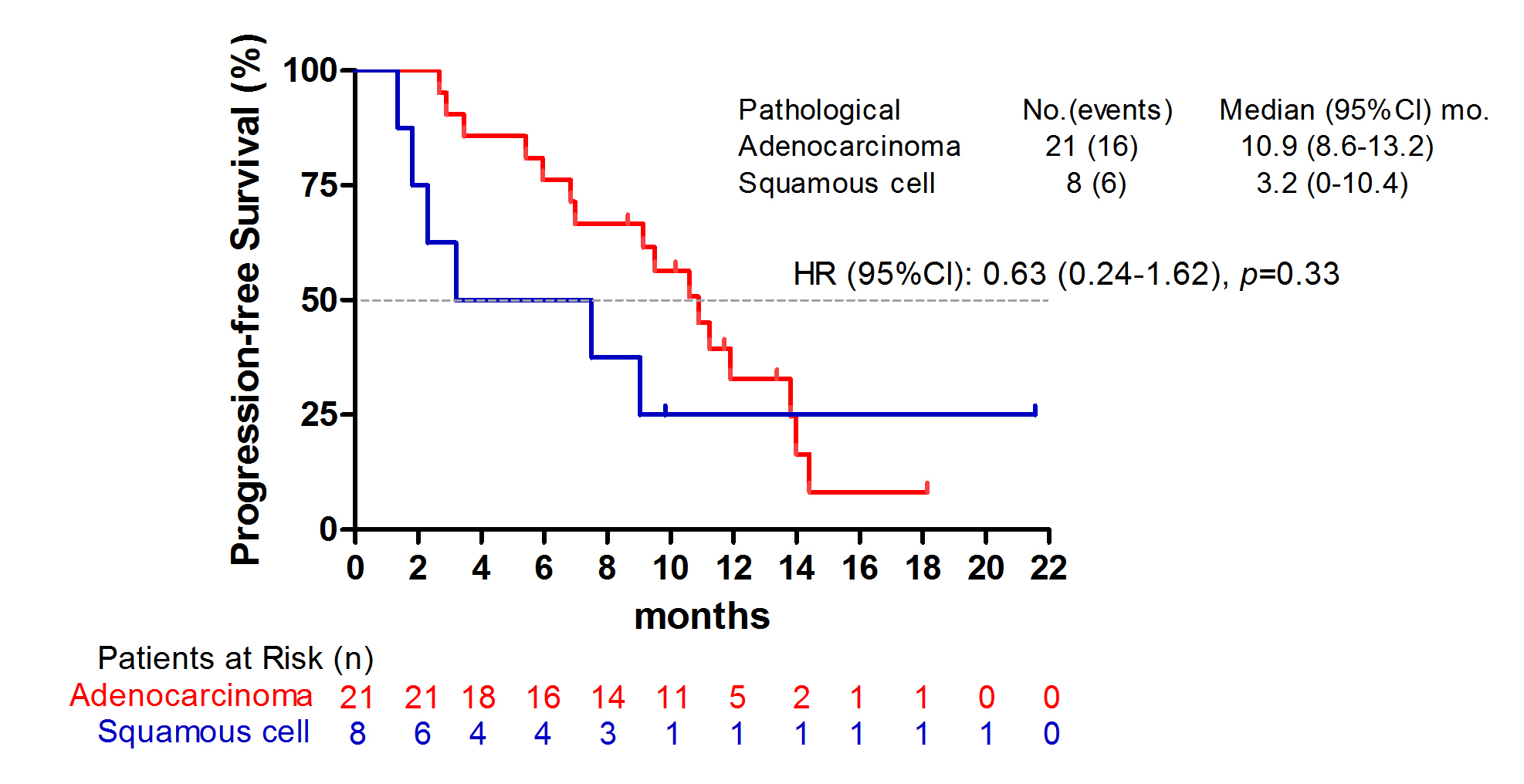


**Figure S1.** Progression-free survival in patients with adenocarcinoma (n = 21) versus in those with squamous cell carcinoma (n = 8). HR, hazard ratio; CI, confidence interval.


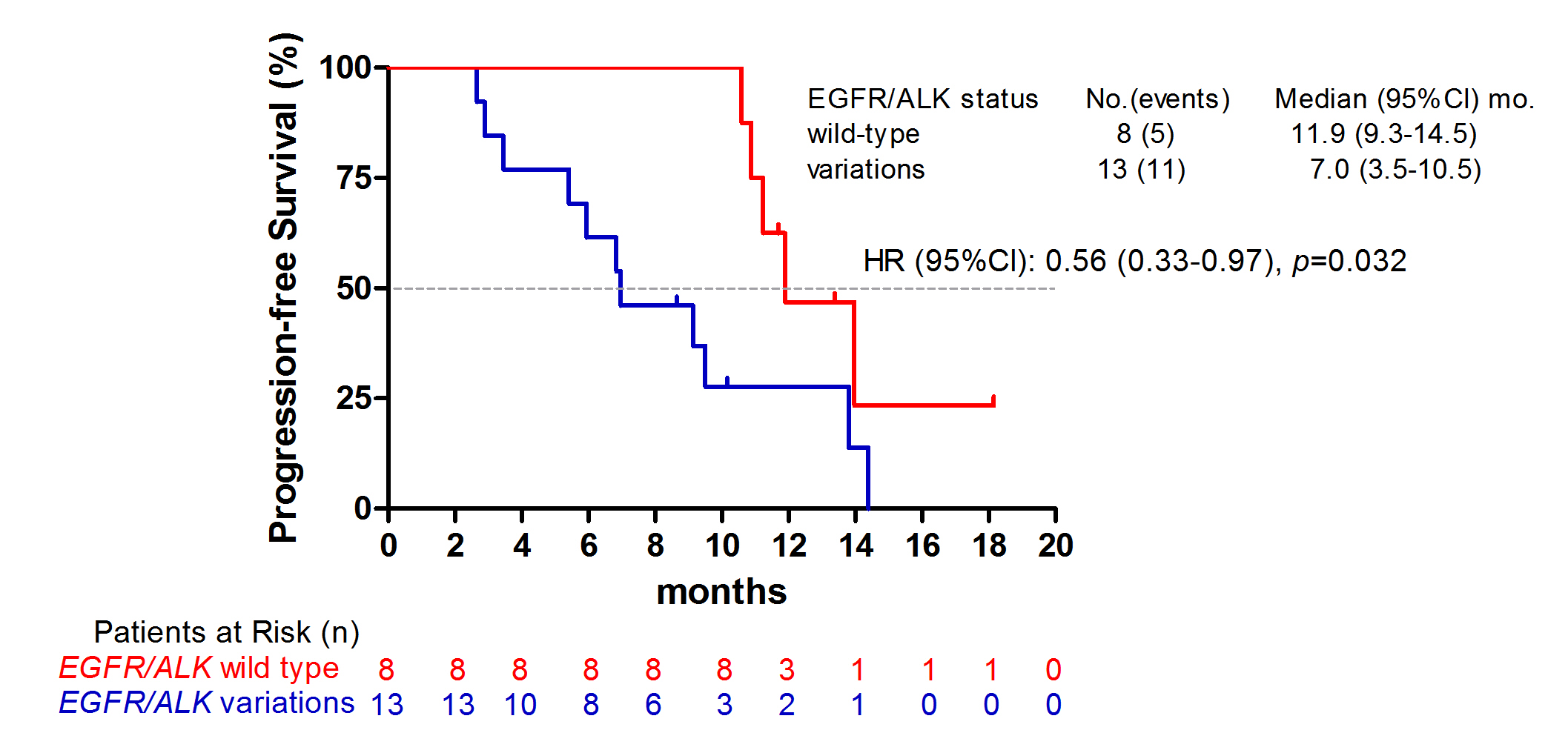


**Figure S2.** Progression-free survival in patients with nonsquamous NSCLC with *EGFR/ALK* wild type (n = 8) versus in those with *EGFR/ALK* variations (n = 13). HR, hazard ratio; CI, confidence interval.
