# Supplementary material for: Efficacy and Safety of Low-Dose Nab-Paclitaxel Plus Tislelizumab in Elderly Patients With Previously Treated Metastatic Non-Small Cell Lung Cancer
Source: Front Oncol. 2022 Mar 17;12:802467. doi: 10.3389/fonc.2022.802467 (PMC8968868; doi:10.3389/fonc.2022.802467)
Supplement: Supplementary file 2 [file Table_1.docx]

**Table S1.** Univariate analysis of factors related to PFS and OS.

| **Subgroup** | **Events, n** | **Median PFS (95% CI)** | ***P* value** | **Events, n** | **Median OS (95% CI)** | ***P* value** |
| --- | --- | --- | --- | --- | --- | --- |
| Age, years | | | | | | |
| <70 (n = 13) | 11 | 6.8 (2.7-10.9) | 0.105 | 7 | 12.7 (4.0-21.5) | 0.355 |
| ≥70 (n = 16) | 11 | 10.6 (7.1-14.0) |  | 6 | NR |  |
| Sex | | | | | | |
| Female (n = 15) | 10 | 10.9 (5.4-16.4) | 0.306 | 6 | NR | 0.480 |
| Male (n = 14) | 12 | 9.0 (5.1-13.0) |  | 7 | 16.4 (6.9-25.8) |  |
| Eastern Cooperative Oncology Group PS | | | | | | |
| 0-1 (n = 12) | 9 | 9.0 (4.1-14.0) | 0.912 | 4 | NR | 0.572 |
| 2 (n = 17) | 13 | 9.5 (4.9-14.1) |  | 9 | 16.4 (11.3-21.5) |  |
| Pathological type | | | | | | |
| Squamous cell carcinoma (n = 8) | 6 | 3.2 (0-10.4) | 0.330 | 5 | 8.1 (2.0-14.1) | 0.076 |
| Adenocarcinoma (n = 21) | 16 | 10.9(8.6–13.2) |  | 8 | NR |  |
| Smoking status | | | | | | |
| Never (n = 17) | 12 | 9.5 (4.9-14.2) | 0.703 | 8 | 16.5 (10.9-22.2) | 0.921 |
| Current/former (n = 12) | 10 | 9.1 (6.7-11.5) |  | 5 | 16.4 (1.5-31.3) |  |
| Oncotarget variation | | | | | | |
| *EGFR/ALK* variations (n = 13) | 11 | 7.0 (3.5-10.5) | 0.191 | 7 | 16.4 (11.5-21.2) | 0.653 |
| *EGFR/ALK* wild type (n = 16) | 11 | 10.9 (9.7-12.1) |  | 6 | NR |  |
| Brain metastases | | | | | | |
| Yes (n = 8) | 6 | 7.5 (2.3-12.7) | 0.912 | 5 | 10.3 (2.5-18.0) | 0.316 |
| No (n = 21) | 16 | 10.6 (8.3-12.9) |  | 8 | NR |  |
| Previous lines of therapy | | | | | | |
| 1 (n = 13) | 10 | 11.2 (8.8-13.7) | 0.176 | 3 | NR | 0.004 |
| ≥2 (n = 16) | 12 | 5.4 (0.5-10.3) |  | 10 | 8.1 (0.9-15.2) |  |

PFS, progression-free survival; OS, overall survival; CI, confidence interval; PS, performance status; NR, not reached.
